# Practice Efficiency in Dermatology: Enhancing Quality of Care and Physician Well-Being

**DOI:** 10.7759/cureus.39195

**Published:** 2023-05-18

**Authors:** Rajani Katta, Emily Strouphauer, Marina K Ibraheim, Jennifer Li-Wang, Harry Dao

**Affiliations:** 1 Internal Medicine, Baylor College of Medicine, Houston, USA; 2 Dermatology, University of Texas Health Science Center at Houston, Houston, USA; 3 Dermatology, Baylor College of Medicine, Houston, USA; 4 Dermatology, Loma Linda University Health, Loma Linda, USA; 5 Dermatology, Katta Dermatology, Houston, USA

**Keywords:** quality of care, physician satisfaction, patient communication, documentation, efficiency

## Abstract

A focus on improved efficiency can impact both patient care and physician well-being. Efficiency is one of the six domains of healthcare quality. It is also recognized as one of the three main pillars of professional fulfillment. Quality improvement measures in the area of efficiency are focused on reducing waste, specifically related to physicians' time, energy, and cognitive demands. Interventions and practices reported in the literature or communicated by dermatologists have documented efforts centered on patient care workflows, documentation, communication, and other areas. Team-based care models maximize the skill sets of other trained providers, while workflow changes encompassing process standardization, communication, and task automatization have improved patient safety and efficiency. Strategies to promote documentation efficiency have centered on eliminating extraneous documentation alongside the use of templates, text expander functionality, and dictation tools. The use of in-office or virtual scribes, when provided with adequate training and consistent feedback, has improved charting time, accuracy, and physician satisfaction. Although upfront investments in time and financial resources may be required, quality improvement in efficiency can benefit healthcare quality, patient safety, and physician satisfaction.

## Introduction and background

Healthcare has been described as a highly complex industry in which complexity is increasing quickly [1]. This has led to multiple challenges, which together threaten the delivery of consistent, high-quality patient care as well as the viability of the healthcare workforce.

A focus on efficiency can improve both patient care and promote the well-being of the healthcare workforce. Efficiency is one of the six domains of healthcare quality as outlined in 1999 by the Institute of Medicine. Efficiency "is about making the best use of resources to promote patient health" [2]. Efficient care avoids waste, including "waste of equipment, supplies, ideas, and energy" [3]. Currently, most quality measures address the domains of safety and effectiveness, while very few assess efficiency [3]. Effective care is defined as the provision of services based on scientific knowledge to all who could benefit while avoiding misuse [3]. The other major quality domains are patient-centered, equitable, and timely care.

Beyond impacts on quality of care, a focus on efficiency can also promote physician satisfaction and fulfillment. According to the Stanford model of professional fulfillment, efficiency of practice is one of the three main pillars of professional fulfillment, alongside a culture of wellness and personal resilience [4]. Attention to dermatologist satisfaction is important and urgent. Between 2011 and 2014, dermatology had the highest increase in burnout prevalence of all specialties [5], and 33% of dermatologists reported burnout in the 2022 Medscape National Physician Burnout and Depression survey [6]. A focus on efficiency can help improve physician satisfaction as it increases the quality, safety, and effectiveness of care while at the same time promoting positive patient interactions and work-life balance [4].

## Review

Quality improvement in efficiency

Quality improvement (QI) is "focused on continuous improvement to improve outcomes" [7]. Quality improvement in efficiency may encompass multiple facets of patient care, from clinic workflows to communication to documentation. A review article by Marsch et al. describes in detail the principles and science of QI and patient safety in dermatology [7] while the American Board of Dermatology provides a range of focused QI projects [8]. Examples of QI projects include interventions that standardize biopsy processes, improve communication, and automate tasks. These have led to reduced medical errors and diagnostic turnaround times, improving patient safety, effectiveness, and efficiency [7,9,10].

In determining areas for quality improvement in efficiency (QIE) that can improve physician satisfaction, reviewing the physician's task load is useful. This may be measured using the National Aeronautics and Space Administration (NASA) task load index (TLX) [1]. To determine the site-specific TLX, physicians are asked to indicate their perceived work demands in four domains: the mental demands, physical demands, time, and effort of workloads. In a nationally representative survey of physicians, the task load score was strongly correlated with burnout, independent of age, specialty, and hours worked [1].

Reducing physician task load, therefore, may help mitigate professional burnout. Notably, this study found a high degree of variation in task load across sites, indicating that some specialties and clinical sites may have best practices worth evaluating further [1].

For QIE targets, measures that reduce physicians’ time, energy, and cognitive demands should be considered. As Shipman (&) Sinsky write, it is important to identify sources of "wasted time", specifically "time spent on activities not requiring physician expertise or not adding value to the patient" [11]. To minimize wasted time and limit procrastination, systems must be designed and continuously improved to optimize the role of physicians. Focusing on time expenditures is important and urgent, as current practices may not be sustainable. In a study of how physicians in ambulatory care spend their time, researchers found that for every hour spent providing direct patient-facing clinical care, two additional hours were spent on electronic health record (EHR) and desk work during the clinic day. Physicians spent another one to two hours at night on clerical work [12].

In addition to time, systems must be redesigned to protect physicians’ cognitive resources. Privitera, Director of the Medical Faculty and Clinician Wellness Program at the University of Rochester, writes: "Brain executive function of the clinician is why patients come to see us…therefore a key organizational intervention is to decrease extraneous cognitive load on clinicians that depletes available executive function" [13].

Some cognitive load is intrinsic to the role of the physician, such as decision-making, diagnosing, and affective labor. In contrast, cognitive load that is extraneous can be minimized by redesigning systems to be more efficient. Examples of tasks that increase extraneous cognitive load include prior authorizations, documentation of quality metrics [14], and non-standardized EHR systems [11]. These tasks represent areas that would benefit from QIE interventions.

Creating durable improvements in efficiency will involve an upfront investment of time and resources combined with a critical evaluation of current workplace systems. A review of successful QIE efforts, as outlined in this article, can be a helpful starting point. Interventions and practices reported in the literature or communicated by dermatologists have documented QIE efforts that are centered on patient care workflows, documentation, communication, and other areas.

Team-based care

One method of reducing time, energy, and cognitive burdens on dermatologists is the use of team-based care models that maximize the skillsets of other trained healthcare providers. These care models can be optimized with enhanced training, standardized workflows, and care protocols.

Medical assistants (MAs) may assist with pre-visit planning, fulfillment of quality metrics, documentation, order entry, and prescription management [15,16]. One dermatology practice uses detailed note templates for specific diagnoses to guide MAs in expanded history-taking and documentation [17].

Some centers have created new positions. Physician partners, such as bachelor’s degree-level personnel, helped facilitate patient care during office visits, resulting in shorter physician time pre- and post-session, as well as shorter patient visits and higher patient satisfaction [18].

Although team-based care may require an upfront investment, there may be significant benefits from improved efficiency. The University of Mississippi's Department of Dermatology conducted an eight-month experiment, in which an additional assistant was hired for a single faculty member. Results included a 30% increase in completed visits and a 33% increase in gross payments, along with improved faculty satisfaction and less at-home charting time. The additional salary costs were covered in just four months [19].

Team-based care models, while effective, must be continuously evaluated. Regular training and the development of standardized processes are necessary to ensure high-quality care from all team members. The scope of practice limitations for medical assistants and other healthcare team members is another important area; these are regulated at the state level and must be considered when developing efficiency strategies.

Another notable challenge during the pandemic has been worker shortages. Appropriate and stable levels of staffing are associated with enhanced physician satisfaction and decreased burnout [20]. However, an overall reduction in the workforce has occurred due to multiple factors, including fear of illness, early retirement, excess deaths, a loss of working mothers from the workforce, and other factors [21]. This has emphasized the importance of hiring and cross-training "front" and "back" office staff to assist with both administrative and clinical duties. Standardization of training processes, with attention to both systems and staff involved in training, is also important to ensure ongoing high-quality and efficient care. Finally, contingency plans for worker absences are critical.

Workflows

Another area of focus is workflows. At the Mayo Clinic's Department of Dermatology, root cause analysis identified several factors that contributed to skin specimen errors [10]. An intervention focused on workflow and technology changes was then instituted. The most impactful was the use of a standard operating procedure displayed in patient rooms that defined the roles of the team members during specimen collection. Another intervention replaced 4000 free-text anatomic site descriptions with 400 standardized anatomic sites. New software was configured to automatically link the biopsy order with label generation. These efforts-encompassing process standardization, communication, and task automatization significantly reduced errors while improving efficiency [10].

At the University of California, San Francisco (UCSF) Department of Dermatology, two wrong-site surgeries prompted a QI project to improve effective biopsy-site photographs. Staff education and posters in clinical workspaces highlighted the criteria for high-quality photos: marked lesion, magnifying photo, and mapping photo [22]. Median high-quality biopsy-site photo rates increased from 59% to 83%.

At Loma Linda University's Department of Dermatology, a QI project instituted by one of the authors (HD) established a protocol for communicating skin cancer results and scheduling surgery. Previously, the time from the release of skin biopsy results to the first attempted contact for surgery scheduling averaged four to six weeks. The prior workflow included paper biopsy results and the involvement of multiple staff members. The revamped process includes a written protocol with expectations for physicians and ancillary staff. For patients with portal access, benign results are released via templated actions. For patients with skin cancer, result notices are routed to the surgery scheduler, who follows a defined protocol: first call attempt within three business days, with the next attempt in one week, followed by a mailed letter, and then a certified letter to the patient. This protocol provides documentation of patient communication efforts and improves the patient experience as well as medicolegal compliance.

Technology, when used optimally, is another important tool in clinic workflows. One academic dermatology center created an electronic handover system to handle tasks that required follow-up, such as the communication of test results. Handover entries in the EHR included a description of the intended plan based on pathology results, streamlining follow-up tasks [23]. As the use of teledermatology increases, studies of best practices regarding how it may be incorporated into practice and its contribution to enhanced efficiency will be important.

Even simple changes to technology or clinic design may reduce the wastage of time. In aggregate, these may have significant benefits. In one study, implementing rapid EHR logins using "tap and go" technology resulted in 56 hours per year of physician time saved [24]. Another study found that a printer in every exam room would save 20 minutes daily per physician [11]. Simply adhering to established protocols, such as scheduling templates and the avoidance of "overbooking" patients, can improve clinic efficiency. The American Academy of Dermatology (AAD) has published best practices for clinic design to enhance efficiency and patient experience [25].

Administrative tasks

Administrative tasks such as pre-authorizations are increasingly prevalent and burdensome [14]. One approach is the use of templates. The AAD offers a customizable pre-authorization denial letter, available for multiple dermatologic drugs and diseases [26]. The work of pre-authorizations may also be consolidated with a staff member who has developed specific expertise.

On a macro level, advocacy for legislative and regulatory solutions must continue. For example, private insurers as well as state Medicaid formularies require pre-authorizations even for many clinically indicated generic medications [27]. In dermatology, the iPLEDGE (FDA, Silver Spring, MD, USA) rollout on a new platform in 2021 resulted in long wait times and added to physician administrative time, as the ancillary staff was no longer granted access to register patients [28].

Documentation

Documentation continues to pose a burden for physicians. One survey demonstrated that physicians spent an average of 44% of their computer-facing time on documentation and clerical tasks, 24% on inbox management, and the remainder on medical care [29]. Strategies to promote efficiency in documentation have centered on elimination, automation, and teamwork. Training in these strategies and others, such as three-day courses, has been linked to improved efficiency, quality, and job satisfaction [30,31].

Targeting "note bloat", with a focus on eliminating extraneous documentation, is one key strategy. As Drummond emphasizes, every component of a note should serve a billing, medicolegal, or continuity of patient care purpose; otherwise, that information should not be included in the patient note [32]. One study found that patient notes in the United States are roughly three to five times longer than those of other countries, pointing to extraneous billing requirements as a root cause [33]. In 2021, changes to outpatient evaluation and management coding were instituted to simplify billing-related documentation. Although physicians must still document clinical care appropriately, documentation of the history of the present illness, review of systems, and examination are not required for billing purposes [34].

Automation, another key strategy, encompasses the use of multiple tools. Template and text expander functionality are found within EHRs. Although the creation of these requires an upfront investment of time, their use can significantly speed up the process of documentation [17]. Dictation tools are also valuable, ranging from traditional dictation services in which an operator transcribes notes to newer digital dictation systems that use machine learning to improve accuracy over time.

Scribes, also known as documentation assistants, are an important component of team-based care in many practices as they reduce the clerical burden on dermatologists. Scribing may be performed by trained MAs, professional in-person scribes, or remote scribes [35]. The Joint Commission does not expressly support or prohibit the use of scribes but does clarify that these may be unlicensed, licensed, or certified individuals who should be trained on medical terminology, principles of billing, EHR functionality, and applicable regulations [35].

In multiple studies, the use of scribes, both in-person and virtual, has been correlated with improved physician satisfaction. One study found that scribes significantly improved overall physician satisfaction with the clinic (OR=10.75), face-to-face time with patients (OR=3.71), charting time (OR=86.09), and accuracy (OR=4.61) [36]. By assuming the responsibility of documenting clinical encounters, scribes grant dermatologists more distraction-free, face-to-face time with patients [37].

In one center, however, it was noted that scribe-documented encounters had longer medical record closure times and after-hour medical record completion, possibly due to the need for physician proofreading [38]. This highlights the need for adequate scribe training and consistent feedback to improve accuracy. Another model utilized by one of the authors (RK) is that of the flipped scribe model. In this model, the physician digitally dictates the note during the visit, following which the scribe completes proofreading and then returns the note for final approval by the physician.

In each of these strategies, a few key points must be emphasized. An initial investment of time is required to train staff, build templates, and learn how to optimize the use of technology tools. One method to optimize technology use is to "outsource the learning curve." This may involve shadowing an electronic medical record (EMR) "superuser" [32] or assigning a staff member to learn the system and then train colleagues. Regardless of the specific methods used, in all instances, close attention must be paid to patient confidentiality.

Patient communication

Patient communication is an increasingly complex area. The Health Information Technology and Clinical Health Act of 2009, the 21st Century Cures Act, and the Merit-Based Incentive Payment System (MIPS) altered clinical practice across the United States by prompting the implementation of the EHR and the patient portal [39-41]. With these tools, patients may be able to reschedule visits, request refills, and solicit clinical advice electronically. Additionally, clinicians who opt into MIPS must provide patients with certain laboratory or pathology results within four business days, with state- and institution-specific interpretations [40,41].

While the patient portal augments the doctor-patient relationship, it can promote burnout by increasing task load and uncompensated documentation time [37,42-46]. Dermatologists receive 49 messages per day on average and 7.8 images from patients per 100 messages; this volume has outpaced time spent in face-to-face clinic encounters [44-46]. Notably, the top 10% of patients who utilize the patient portal account for 41% of the message requests [44].

Dermatologists can employ several tactics to optimally manage the patient portal and convert it into a tool that delivers high-quality patient care. Similar to documentation efficiency, strategies to promote efficiency may focus on elimination (or reduction), automation, and teamwork (see Table 1).

**Table 1:** Efficiency strategies for patient communication EMR: Electronic medical record

Strategy	Examples
Reduction	Staff phone call within two days after Mohs surgery to preemptively address likely patient-initiated communication
	Expand postoperative instructions to proactively address common questions
	Expand postoperative instructions to include photographs of expected healing to address common questions on postsurgical healing
	New policies that clarify which patient portal messages are considered virtual healthcare and thus will be billed, versus other types of messages that will not be billed (e.g., prescription refills)
Automation	Create templates for laboratory and pathology results to expedite the dissemination of result notes to patients
	Use text expander functionality in EMR to provide scripted responses to patient questions
	Scripted responses may include links (e.g., online resources, videos, referred physician websites)
Teamwork	Utilize message pools: incoming messages are assigned to a pool of staff members to facilitate cross-coverage and streamline the delegation process for physicians
	Support staff assigned to triage messages; primarily non-urgent medical management questions are forwarded to the clinician
	Urgent questions answered over the phone
	Written protocols and clearly defined expectations
	Consider the state-specific scope of practice regulations regarding message triage and delegated actions
	Document all instructions to staff and efforts to message patients

In one prospective trial, a simple staff phone call within one to two days after Mohs micrographic surgery resulted in fewer patients initiating contact post-procedure [47]. As Mazmudar et al. state, "preemptively addressing likely episodes of patient-initiated communication . . . may help free up valuable clinic resources for direct patient care."

One academic dermatologic surgery clinic sought to design a QI intervention that would reduce both incoming messages and physician participation in these messages [48]. They investigated portal message encounters to identify common themes and then designed bundled interventions. The QI changes were assessed post-intervention and documented fewer messages with direct replies from physicians. Interventions included scripting responses to common patient portal medical questions and delegating these to team members. Postoperative instructions were also expanded to address common questions and included photographs of expected healing to address common questions related to postsurgical healing.

The use of message pools, in which incoming messages are assigned to a pool of staff members, has multiple benefits. At the Loma Linda University Department of Dermatology, this decreases the burden on individual staff members and facilitates cross-coverage in case of vacation or sick days. In addition, this streamlines the delegation process for physicians. Separate pools are used for communicating non-urgent and urgent messages, each with its own written protocol and expectations. This system also provides metrics on the number of messages handled for each ancillary staff member, allowing for productivity measures and allocation of staff administrative time. In addition, clinic managers have real-time oversight of the message pools to better identify areas needing attention.

Strategies shared by dermatologists reflect additional approaches. In a contact dermatitis practice, one of the authors (RK) created extensive patient education handouts and scripted templates for staff members to use in response to common patient questions. Houk outlined several of her successful strategies in an interview. First, support staff can triage messages and reserve non-urgent medical management questions for the clinician. Urgent questions are better addressed over the phone. Second, creating templates tailored to laboratory or pathology results can expedite the dissemination of result notes to patients. Additionally, establishing expectations with patients regarding result note dissemination may minimize distress precipitated by communication delays [40,49].

In implementing these strategies, dermatologists must consider the state-specific scope of practice regulations that may apply to message triaging or delegated actions. All instructions to staff must be documented, and staff should likewise document their efforts in messaging patients for medicolegal purposes so that colleagues can cross-cover with less repeated work.

Finally, reimbursement for virtual patient communication must be considered. Both UCSF [50], as of November 2021, and the Cleveland Clinic [51], as of November 2022, have instituted new policies clarifying that portal messages that require providers’ medical expertise and that take longer to answer is considered a form of virtual healthcare ("e-visits") and will be billed as such. Other types of messages, such as prescription refills, will not be billed. Following policy implementation at UCSF, a reduction in patient portal messaging was noted, although e-visit adoption was low [52].

## Conclusions

With the rise of new technologies, medical practice regulations, changing patient expectations, and even global pandemics, dermatologists must become adept at modifying their workflows to respond to new challenges. Such challenges will continue to impact the practice of medicine. The implementation of QIE projects that reduce wasted resources, specifically dermatologists’ time, energy, and cognitive resources, is valuable. Beyond benefits to dermatologists, this focus can produce benefits in other domains, including healthcare quality, patient safety, and financial revenue, even if upfront investments in time and financial resources are required. As further QIE projects are developed, tested, and shared, the outcomes and lessons learned can provide inspiration and valuable guidance.
